# Performance of synacthen test in chronic hemodialysis patients

**DOI:** 10.1186/s12882-023-03347-3

**Published:** 2023-11-07

**Authors:** Manuela Nickler, Susanne Stampf, Luca Bernasconi, Stephan Segerer, Claudine A. Blum, Min Jeong Kim

**Affiliations:** 1 Medical University Department, Division of Nephrology, Kantonsspital Aarau, Aarau, Switzerland; 2 Clinic for Transplantation Immunology and Nephrology, University Hospital Basel, Basel, Switzerland; 3 Center for Laboratory Medicine, Institut of Immunology & Clinical Chemistry, Kantonsspital Aarau, Aarau, Switzerland; 4 Hormonpraxis Aarau, Aarau, Switzerland; 5 University of Basel, Basel, Switzerland

**Keywords:** Adrenal insufficiency, Hemodialysis, Synacthen test, Cortisol, Equivalence

## Abstract

**Background:**

Adrenal function tests (Synacthen test) in chronic hemodialysis (HD) patients are currently performed off dialysis. The study aimed to demonstrate equivalence of serum cortisol concentrations pre- and during HD, each for standard-dose (250 µg) and low-dose (1 µg) Synacthen test.

**Methods:**

In a single-center cross-over diagnostic equivalence study, Synacthen tests were performed in four settings, in standard- and low-dose as well as pre- and during HD. Serum cortisol concentration was measured at 30 and 60 min after Synacthen administration, and additionally at 20 min in low dose test. Based on a multivariable linear mixed model the means of cortisol concentration on log-scale were estimated in each dose and test time combination. Differences in means were calculated and the TOST approach was applied to test for equivalence. Equivalence was proven if the 90% confidence interval of the difference of two cortisol means was entirely between − 0.22 and 0.22.

**Results:**

In 28 chronic HD patients, serum cortisol concentrations at 30 and 60 min after Synacthen administration in both standard- and low-dose were shown to be equivalent pre- and during HD. In 10 of 56 low-dose tests, the cortisol peak was already reached after 20 min. However, cortisol concentrations at 20 and 30 min after low-dose Synacthen test pre- and during HD showed no significant difference.

**Conclusion:**

These results suggest that the adrenal function test may be carried out during an ongoing HD session, leading to a more patient-friendly performance of the test, less organizational effort and potentially earlier diagnosis of adrenal insufficiency.

**Supplementary Information:**

The online version contains supplementary material available at 10.1186/s12882-023-03347-3.

## Introduction

Adrenal insufficiency is a potentially life-threatening condition due to the risk of adrenal crisis, especially during acute illness. The most common cause of adrenal cortical insufficiency is glucocorticoid treatment [1, 2]. Since immunosuppressive therapy after renal transplantation most often includes prednisolone, this patient population is at risk for impaired adrenal function, not only after withdrawal, but also during ongoing low-dose glucocorticoid treatment [3]. A recent case-control study showed that adrenal insufficiency occurred in 43% of patients treated with either 5 or 7.5 mg prednisolone daily after kidney transplantation [3]. In a subgroup of 50 chronic hypotensive patients with end-stage kidney disease (ESKD) on dialysis, adrenal insufficiency was found in 20% (8% classified as primary, 12% as secondary adrenal insufficiency) [4]. Thus, increased clinical alertness towards adrenal insufficiency in hemodialysis (HD) patients seems to be crucial.

The Synacthen stimulation test is a simple method to investigate adrenal cortical function [5, 6]. Several observational studies have shown normal adrenal responsiveness to exogenously administered adrenocorticotropin (ACTH) in HD patients compared to patients without renal failure [7–11]. This was demonstrated in ACTH test with standard dose 250-µg as well as with low dose 1 µg, although there was a trend toward a diminished cortisol release after 1 µg in HD patients compared to healthy controls [11]. These data support in general the use of Synacthen test in HD patients. However, tests in the previous studies have been performed off dialysis, and less is known about the effect of ongoing HD on the response to exogenously administered ACTH [12]. Due to the above-mentioned high prevalence of adrenal insufficiency among HD patients, the increasing number of patients returning to dialysis after renal transplantation and also patients with ongoing need for renal replacement therapy after acute kidney injury in the intensive care setting, the demand for Synacthen test in the HD patient population is expected to be increasing. Performing the test during a dialysis session could therefore significantly reduce additional organisational efforts and is more patient-friendly.

We therefore aimed to show equivalence of Synacthen test performed pre- and during HD session. Further, we studied the within-patient variation of cortisol concentrations during an HD session without Synacthen administration.

## Method

### Study design

The study was designed as a single-center cross-over study to show equivalence of Synacthen test performed pre- and during HD. The study protocol and informed consent form were approved by the ethics committee of the northwest and central Switzerland (Reference number 2020–02157).

### Study aims and outcome definition

The study outcome was the individual serum cortisol concentration at defined time points after stimulation with standard-dose Synacthen (250 µg) and low-dose Synacthen (1 µg) pre- and during HD. For each Synacthen dose, the equivalence of cortisol concentration pre- and during HD was primarily aimed to prove at 30 and 60 min, respectively. Further study aims were to study the variation of serum cortisol concentration during an HD session without Synacthen stimulation and to detect the peak of cortisol concentration after low-dose Synacthen stimulation.

### Patient selection

Patients older than 18 years with ESKD receiving chronic HD on a thrice-weekly 4-hour HD schedule who provided written informed consent were included in our study. Exclusion criteria were ongoing glucocorticoid treatment or glucocorticoid treatment in the previous 12 months for 2 weeks or longer, known adrenal insufficiency, acute illness or hospital admission during the last 4 weeks, pregnancy and ongoing treatment with oral contraception.

### Study conduct

For each study participant we planned a five-week study schedule (supplementary Fig. 1). Synacthen tests and laboratory measurements were always carried out at the second HD of the week with a minimum one-week interval between the tests, while keeping the participants’ usual HD starting time either in the morning or afternoon. Standard-dose Synacthen (250 µg) was dissolved in 1 ml fluid containing acetic acid, sodium acetate, sodium chloride and water, and was directly injected intravenously. To administer low-dose Synacthen (1 µg), dilution with additional 0.9% sodium chloride (at 1 µg/ml) occurred with subsequently direct intravenous injection.

On the first day of the study, blood sampling at defined time points during an HD without Synacthen administration was performed (cortisol profile). Serum cortisol, glucose and hematocrit were measured at the beginning of HD, 30 and 60 min afterwards and at the end of HD. Serum albumin was measured only at the start of HD. On each of the four subsequent study days, one Synacthen test per day was performed according to the randomly assigned test schedule. We defined four different settings, i.e. standard-dose Synacthen (A - before HD, B - during HD) and low-dose Synacthen administration (C – before HD, D - during HD). Synacthen injection took place immediately after dialysis start. Blood samples immediately before, and 30 and 60 min after Synacthen injection for the measurements of cortisol, glucose and hematocrit were collected at each study day. In case of low-dose Synacthen administration, an additional blood sample 20 min after Synacthen injection was taken. During the Synacthen test (approximately an hour time period) patients were not allowed to consume food or beverage, and the ultrafiltration rate and blood flow were kept as constant as possible.

### Laboratory measurements

Blood samples were collected from the arterial line of dialysis access and were stored at -80 °C until analysis. Serum cortisol was measured by a chemiluminescent microparticle immunoassay (CMIA). To exclude a possible effect of volume removal (ultrafiltration) induced intravascular volume change on the cortisol levels, we corrected measured cortisol values during HD for hemoconcentration according to Schneditz et al. [13]. The change in intravascular volume was calculated from the change in hematocrit (($\frac{{H}_{{t}_{0}}}{{H}_{{t}_{x}}}-1)*100$) with ${H}_{{t}_{0}}$ representing the hematocrit at the start of HD and ${H}_{{t}_{x}}$ representing the hematocrit at x minutes thereafter during HD. According to the international guidelines of the endocrine society [14], we defined sufficient adrenocortical responsiveness to Synacthen administration as an increase of serum cortisol concentration above 500 nmol/l 60 min after standard-dose (250 µg) and 30 min after low-dose (1 µg) [15, 16]. Glucose and albumin measurements were performed by an enzymatic test, and hematocrit was calculated using mean corpuscular volume (MCV) and red blood cell count measured by impedance counters.

### Sample size

A total of 28 chronic HD patients were randomly and evenly assigned to one of the four sequences ADBC, BACD, CBDA, DCAB according to Williams’ design [17, 18]. We tested equivalence using the TOST (two one-sided t-tests) approach with significance level of 5% and target power of 80% [19]. Based on previous studies showing variation in within-subject measurements across the different study populations, we assumed a coefficient of variation (CV) estimate of 0.2608 in our study [20]. Two means were considered as equivalent if their ratio is within the equivalence bounds of 80% and 125% [21]. The alternative hypothesis was thus formulated as equivalence of all pairs of measurement protocols and accepted if all pairwise tests were significant at the level of 5%. The sample size for our 4 × 4 design (four measurement protocols, four sequences) was calculated with the R package “PowerTOST” [22].

### Statistical analysis

Demographic and clinical characteristics of the study participant at study entry were descriptively shown by means and frequencies with standard deviation and interquartile range, respectively. We used *generalized (additive) mixed model*s to test our hypothesis of equivalence. The *random intercept* included in the model accounted for the variation in measurements for subjects due to multiple measurements over time. We additionally accounted for patient and HD information, and tested for potential *order and carry-over effects* by including the test sequence and the corresponding study day into the model.

In order to investigate the equivalence of serum cortisol concentrations pre- and during HD, we considered measurements at 30 and 60 min after Synacthen administration. Serum cortisol values shortly before Synacthen were accounted for in the regression model as well as a triple interaction between Synacthen dose (standard or low), measurement time after Synacthen (30 or 60 min after Synacthen) and the measurement setting (pre- and during HD). It allowed to estimate means of cortisol concentrations in each dose and time combination of interest. We further adjusted for potential effects of albumin, glucose and HD daytime. Differences in means were calculated and the TOST approach was applied to test for equivalence in each of the settings. As estimations were made on log-scale, two means were considered as equivalent if their difference is in between − 0.22 and 0.22 being the log-transformed equivalence bounds of 80 and 125%.

Patient’s cortisol profile, i.e. serum cortisol course during HD without Synacthen stimulation, was estimated using cortisol measurements at start, at 30 and 60 min during and at the end of HD. Patient-specific time courses were used to show within-patient variation graphically.

To detect the peak of serum cortisol concentration after low-dose Synacthen, models included cortisol measurements at 20, 30 and 60 min after low-dose Synacthen administration.

In regression models for cortisol profile and low-dose Synacthen data only, we allowed for a flexible non-linear effect of time on cortisol concentration by modelling time via *restricted cubic splines*.

All statistical analyses and visualization were performed using R version 4.1.2 [22]. Generalized additive mixed models were fitted using the R package ‘mgcv’, and ‘lmer’ in case of a linear effect of time. The package ‘ggplot2’ was used for visualization.

## Results

### Clinical and demographic characteristics of study participants at study begin

Among 28 enrolled chronic HD patients, the median age was 70.5 years and 20 patients (71.4%) were male (Table 1). Two study patients had a previous kidney transplantation (7.1%) and five received previous glucocorticoid treatment (17.9%). The most common causes of end stage kidney disease (ESKD) were vascular nephropathy (39.3%) and diabetic kidney disease (35.7%). Almost 90% of the study participants had multiple comorbidities.

**Table 1:** Clinical and demographic characteristics of study participants at study begin

	Total (N = 28)
**Median age in years at study begin (IQR)**	70.50 (63.00, 75.50)
**Gender (male)**	20 (71.4%)
**Caucasian**	25 (89.3%)
**Previous kidney transplantation**	2 (7.1%)
**Previous glucocorticoid treatment ≥ 2weeks (≥ 1year ago)**	5 (17.9%)
**Median HD vintage in months (IQR)**	36.50 (15.50, 77.50)
**Dialysis access: Arteriovenous fistula / graft**	24 (85.7%)
**Cause of end stage kidney disease (ESKD)**	
- Vascular - Diabetes - ADPKD - Glomerulonephritis - Tubulointerstitial disease - Other*/ Unknown	11 (39.3%) 10 (35.7%) 1 (3.6%) 4 (14.3%) 1 (3.6%) 11 (39.3)
**Patients with multiple causes of ESKD**	7 (25%)
**Native kidney biopsy**	10 (35.7%)
**Comorbidities**	
- Coronary artery disease - Congestive heart failure - Hypertension - Peripheral artery occlusive disease - Diabetes mellitus - Obesity - Neoplastic disease - Other**	9 (32.1%) 7 (25.0%) 24 (85.7%) 14 (50.0%) 13 (46.4%) 6 (21.4%) 4 (14.3%) 7 (25.0%)
**Patients with multiple comorbidities**	25 (89.3%)
**Concomitant medication**	
- Loop diuretics - Thiazide diuretics - RAAS inhibitor - CCB blocker - BB - Insulin	17 (60.7%) 7 (25.0%) 10 (35.7%) 6 (21.4%) 18 (64.3%) 7 (25.0%)

Abbreviations: ADPKD – autosomal-dominant polycystic kidney disease; BB – beta blocker; CCB – calcium channel antagonist; HD – HD; IQR – interquartile range; RAAS – renin-angiotensin-aldosterone system

* Other ESKD: nephrectomy (bilateral or unilateral), cardiorenal syndrome, cardiogenic shock, chronic hydronephrosis, acute tubular necrosis due to septic shock, lithium-associated nephropathy, secondary focal segmental glomerulosclerosis, thrombotic microangiopathy due to atypical hemolytic-uremic syndrome (factor H deficiency)

** Other comorbidities: hypertensive heart disease, valvular heart disease, atrial fibrillation, chronic bronchitis, chronic autoimmune thyroiditis, osteoporosis, benign prostatic hyperplasia, polyarthritis, polyneuropathy, idiopathic Parkinson’s disease

### Equivalence of cortisol concentrations after Synacthen administration pre- and during HD

**Fig. 1:**
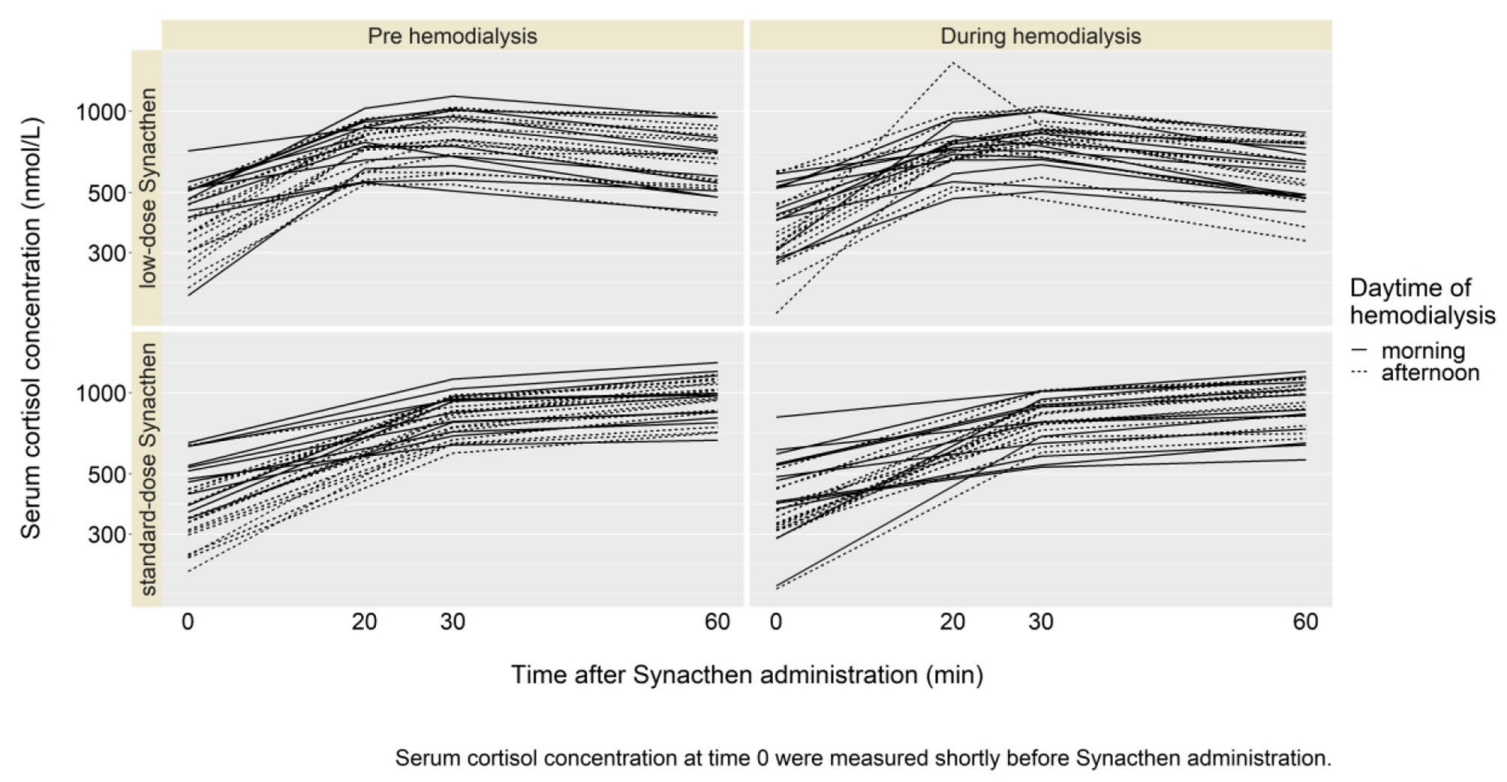
Cortisol concentrations of 28 study participants in the four different settings of Synacthen dose (low-dose (1 µg) and standard-dose (250 µg)) and administration (pre- and during HD. Abbreviation: HD – hemodialysis

**Fig. 2:**
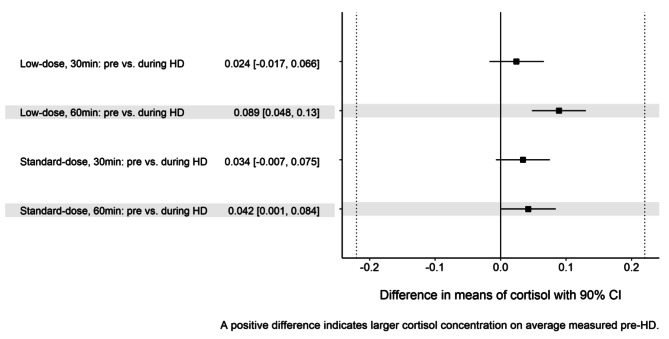
Differences of means of estimated cortisol concentration (nmol/l) on log scale and corresponding 90%–confidence intervals (CI) in each dose and administration time combination at 30 and 60 min after Synacthen. Abbreviation: CI – confidence interval, HD – hemodialysis. The equivalence region is shown by vertical dotted lines. All 90%-CI are entirely included in the equivalence region, equivalence for all settings has been proven

All the participants showed sufficient increase of serum cortisol concentration (> 500 nmol/l) in four different settings, i.e., standard- (250 µg) and low- (1 µg) Synacthen dose as well as pre- and during HD (Fig. 1). Serum cortisol concentrations after 30 and 60 min, respectively after standard- and low-dose Synacthen administration, were found to be equivalent pre- and during HD. All related 90% confidence intervals for differences in mean cortisol concentration on log-scale were within the equivalence bounds (Table 2; Fig. 2). After standard Synacthen dose, a steady increase is observed until 60 min, while after low Synacthen dose, the highest values of cortisol concentration were already reached at 30 min in most cases (Fig. 1). The differences in the estimated mean values of cortisol between pre- and during HD were lower at 30 compared to 60 min, both after the standard and low Synacthen dose (Fig. 2). Neither a carry-over nor an order effect was found in our cross-over study (suppl. Table 1). Cortisol concentration measured shortly before Synacthen administration had a significant effect on the concentration after Synacthen (p < 0.001) (suppl. Tables 1–2). Patients with HD in the afternoon had higher cortisol concentrations after Synacthen than patients having HD in the morning, the difference was however not significant (p = 0.08). Albumin and glucose did not affect significantly cortisol concentration after Synacthen. Cortisol concentration after Synacthen showed a significant variability between study participants (σ_b_ = 0.172, 95%-CI: 0.12–0.2) explaining around 79% of random variability of cortisol induced by patients.

**Table 2:** Equivalence test results for measured cortisol concentration (nmol/l) on log-scale shown in four different Syancthen dose and time settings. Estimates were fitted in a multivariable linear mixed model with random intercepts per subject accounting for multiple measurement at different time points after Synacthen. It was further adjusted for patient and HD variables: cortisol concentration shortly before Synacthen administration, albumin at HD start on study day 1, glucose measured after Synacthen, daytime of HD, test sequence and study day

Synacthen dose	Time after Synacthen administration (min)	Time of Synacthen administration	Mean (SE) of cortisol concentration on log-scale	Difference in means of cortisol concentration on log-scale pre- vs. during HD [90% CI]
Low	30	pre-HD	6.654 (0.0375)	0.024 [-0.017, 0.066]
		during HD	6.630 (0.0373)	
	60	pre-HD	6.474 (0.0373)	0.089 [0.048, 0.130]
		during HD	6.385 (0.0373)	
Standard	30	pre-HD	6.692 (0.0374)	0.034 [-0.007, 0.075]
		during HD	6.658 (0.0373)	
	60	pre-HD	6.838 (0.0374)	0.043 [0.001, 0.084]
		during HD	6.796 (0.0374)	

Abbreviations: CI – confidence interval; HD – hemodialysis; SE – standard error. Means and differences of means of cortisol concentration (nmol/L) on log-scale in each Synacthen dose measured at 30 and 60 min after Synacthen. All 90% confidence intervals are entirely in equivalence region of (-0.22, 0.22). Equivalence of cortisol concentration pre- and during HD at 30 and 60 min after Synacthen was thus proven in all four scenarios

The equivalence tests using cortisol values corrected for hemoconcentration revealed similar results with proven equivalence pre- and during HD (suppl. Table 3).

### Peak cortisol concentration after low-dose Synacthen administration

**Fig. 3:**
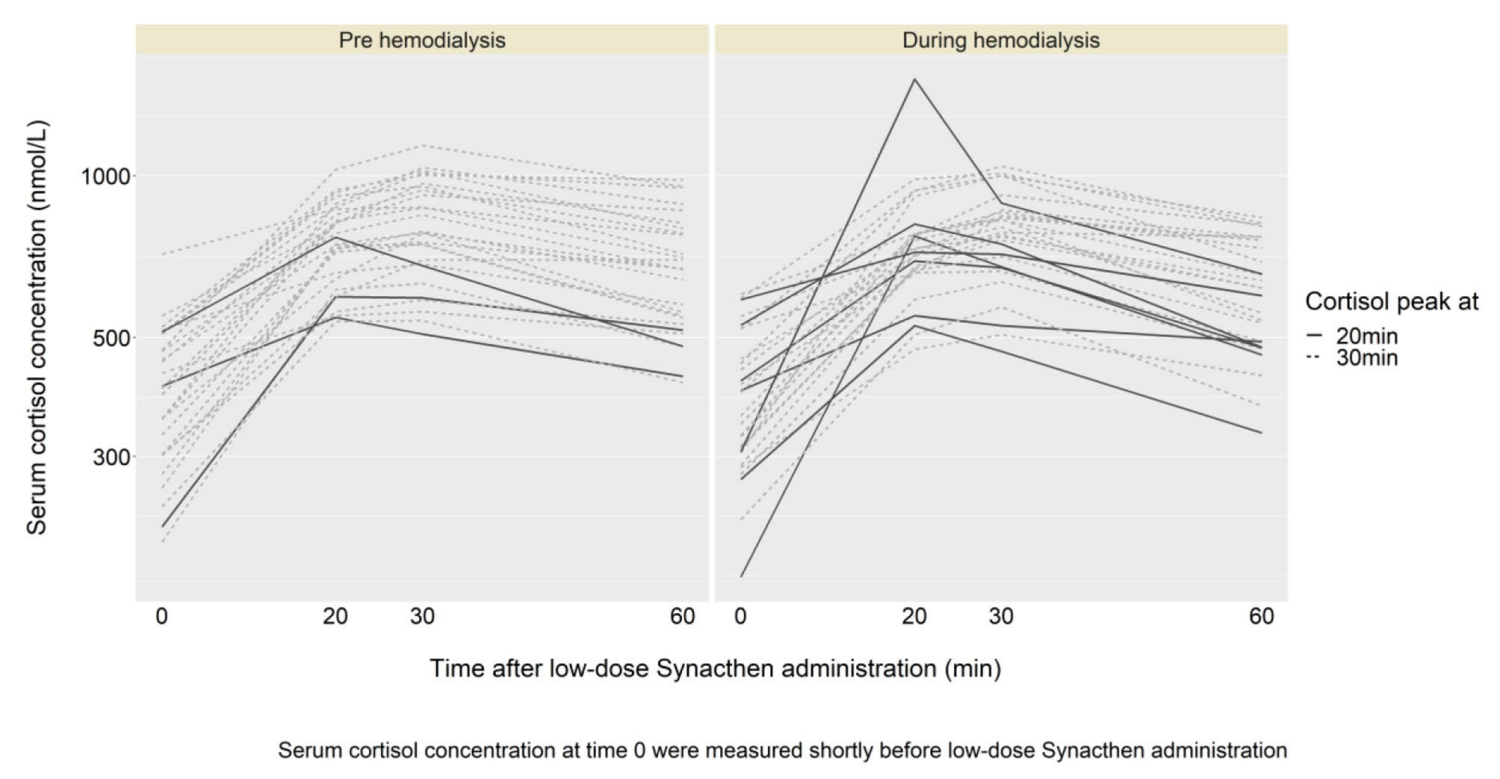
Cortisol concentration (nmol/l) after low-dose Synacthen administration pre- and during HD in the 28 study participants. Abbreviation: HD – hemodialysis

In ten of 56 performed low-dose Synacthen tests, the peak of cortisol concentration was already reached after 20 min, and seven of them were observed during HD (Fig. 3). There was no significant difference between cortisol concentration at 20 and 30 min after low-dose Synacthen test pre- as well as during HD.

### Variation in cortisol concentration during HD without Synacthen stimulation

**Fig. 4:**
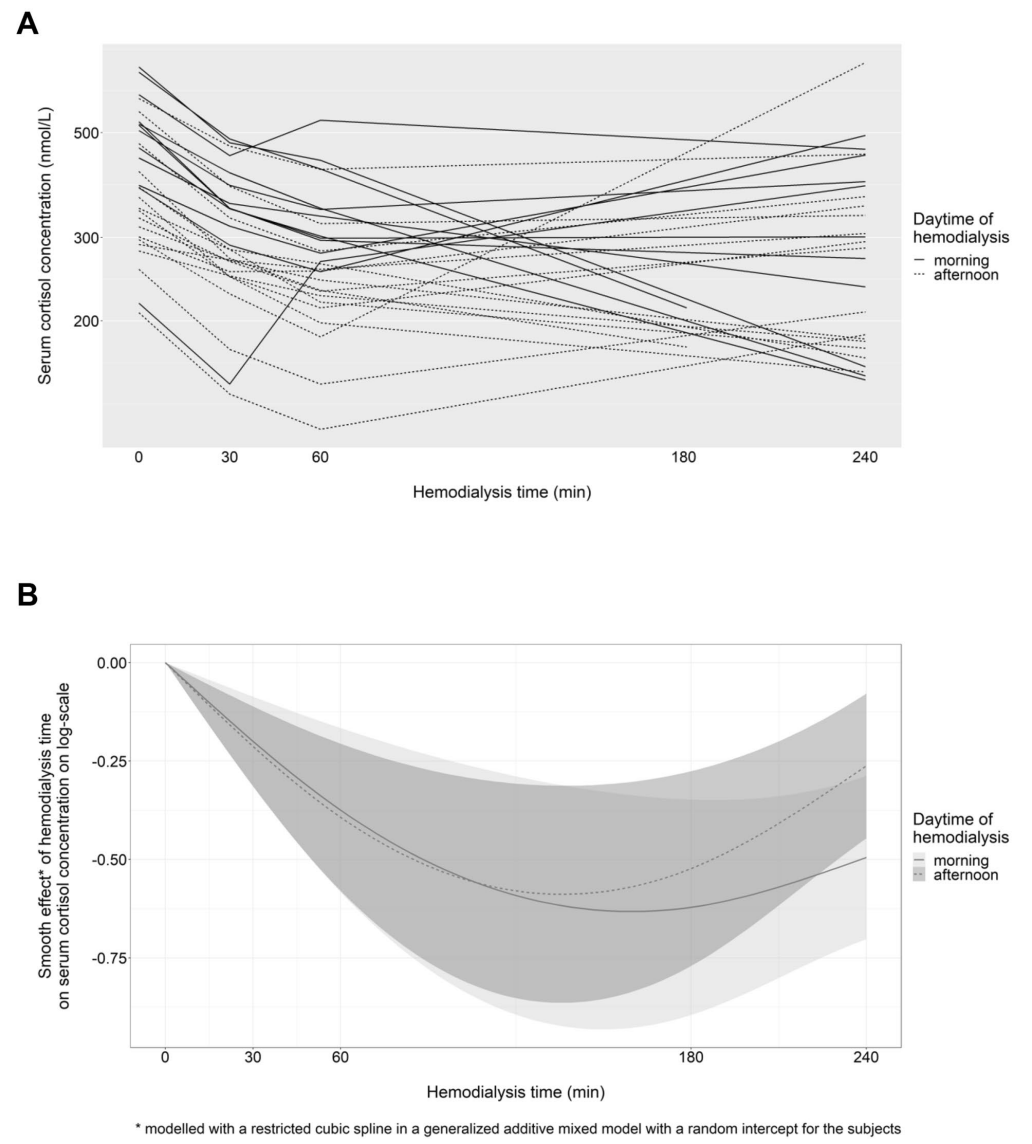
Changes in serum cortisol concentration during HD without Synacthen (**A**) and estimated smooth effects for HD time on serum cortisol concentration (nmol/l) on log-scale from a multivariable linear additive mixed model (**B**). Abbreviation: HD – hemodialysis. The random intercept has accounted for multiple cortisol measurements during HD for each subject and HD time was modelled using restricted cubic splines

A stepwise decrease in cortisol value during the first 60 min after HD start, followed by a slight increase until the end of the HD session was observed in most patients (Fig. 4A). Cortisol levels were often lower in patients with HD in the afternoon, but with a steeper rise at the end of HD than observed in patients with HD in the morning (Fig. 4A and B). These findings were confirmed by fitting a linear additive mixed model, where HD time was flexibly modelled as restricted cubic splines by HD daytime (Fig. 4B; Table 3). In addition, higher systolic blood pressure during HD was significantly associated with lower cortisol concentration. There was no significant association of serum albumin or serum glucose with cortisol concentrations during HD. The between-patient variability was similar as in the model performed for the equivalence study, contrary, the within-patient variability was higher.

**Table 3:** Model results for estimating the cortisol profile during HD in 28 study participants without Synacthen. Estimated effects from univariate models and a final multivariable linear additive mixed model were shown. Multiple measurements during HD per subject were accounted for including a random intercept for subjects. Serum cortisol concentration is modelled on the log-scale accounting for patient and HD variables. Time during HD is assumed to be non-linear and fitted as restricted cubic splines

Effect estimate (95% CI) [p-value]	Univariate	Multivariable
*Patient variables at start of HD*		
**Age (years)**	0.003 (-0.007, 0.013) [0.55]	
**Gender:**		
- Male - Female	(reference) -0.04 (-0.26, 0.18) [0.72]	
**Dry weight (kg)**	0.002 (-0.003, 0.006) [0.48]	
**Albumin (g/l)**	0.008 (-0.02, 0.03) [0.57]	0.009 (-0.01, 0.03) [0.43]
*Patient variables varying over HD time*		
**Loss of weight between start and end of HD (kg)**	-0.04 (-0.12, 0.04) [0.35]	
**Glucose (mmol/l)**	0.007 (-0.02, 0.04) [0.68]	-0.010 (-0.04, 0.02) [0.46]
Systolic blood pressure*	-0.003 (-0.007, -0.0002) **[0.04]**	-0.005 (-0.007, -0.002) **[0.002]**
Diastolic blood pressure*	0.001 (-0.005, 0.006) [0.79]	
**Daytime of HD:**		
- Morning - Afternoon	(reference) -0.27 (-0.44, -0.09) **[0.006]**	(reference) -0.26 (-0.44, -0.09) **[0.005]**
**Ultrafiltration amount**	-0.016 (-0.04, 0.009) [0.021]	
**HD time (min)**		
- 0 - 30 vs. 0 - 60 vs. 0 - 240 vs. 0	(reference) -0.22 (-0.29, -0.14) -0.4 (-0.53, -0.27) -0.35 (-0.49, -0.22)	
	**[< 0.001]**	
**HD time (min) at morning:**		
- 0 - 30 vs. 0 - 60 vs. 0 - 240 vs. 0		(reference) -0.2 (-0.31, -0.09) -0.38 (-0.58, -0.17) -0.50 (-0.70, -0.29)
		**[< 0.001]**
**HD time (min) at afternoon**:		
- 0- 30 vs. 0- 60 vs. 0- 240 vs. 0		(reference)-0.21 (-0.31, -0.11)-0.39 (-0.58, -0.21)-0.26 (-0.45, -0.08)
		**[< 0.001]**
*Random effects*		
**Between-subject variability (σ**_**b**_ **)** **Within-subject variability (σ**_**ε**_ **)**		0.18 (0.11, 0.29) 0.26 (0.21, 0.31)

Abbreviations: CI – confidence interval; HD – HD.* 19 missing values in systolic and diastolic blood pressure measurement

The same analyses using cortisol values corrected for hemoconcentration revealed similar results (supplementary Fig. 2 and supplementary Table 4).

## Discussion

To our knowledge, this is the first study investigating the effect of ongoing HD on the adrenal response to exogenously administered ACTH, providing novel insights into the diagnosis of adrenal insufficiency in chronic HD patients. We found equivalent cortisol concentrations after Synacthen stimulation pre- and during HD for both standard-dose and low-dose Synacthen and for tests in the morning as well as in the afternoon. Several previous observational studies have shown normal adrenal responsiveness to Synacthen stimulation in HD patients off dialysis session compared to patients without renal failure [7–11]. However, besides our study there is so far no other data available investigating the reliability of adrenal function tests during ongoing HD [12].

Serum cortisol concentrations were measured at 30 and 60 min after Synacthen stimulation, and additionally at 20 min after low-dose Synacthen. In a multivariable model, equivalence of cortisol concentrations after Synacthen stimulation pre- and during HD was shown for standard- as well as low-dose Synacthen and for tests in the morning as well as in the afternoon. Interestingly, hemoconcentration during HD had no influence on the equivalence tests, thus the effect of volume removal on cortisol concentrations after Synacthen stimulation seems negligible in our study. Moreover, even though plasma cortisol clearance seems to increase during the first hour of HD ( [23] and our own data), this did not influence the results of Synacthen test (equivalence pre- and during HD). The underlying physiological aspects of the surprisingly negligible effects of volume removal and plasma cortisol clearance on the results of Synacthen test during ongoing HD are still poorly understood and were not the main subject of this study. Further studies with additional measurements of ACTH and free (unbound) cortisol levels are warranted to better understand the underlying physiology. Neither serum glucose, nor serum albumin, nor daytime of HD (morning versus afternoon) altered serum cortisol response to Synacthen.

Previous studies showed the clinical usefulness of low-dose Synacthen test (1 µg) in excluding adrenal insufficiency with higher sensitivity [15, 16, 24]. A previous study showed a trend toward a diminished cortisol release after low-dose (1 µg) ACTH stimulation in HD patients (tests performed off dialysis session) compared to healthy controls [11]. In the present study, we did not have healthy controls, but – defining a serum cortisol concentration cut-off of > 500 nmol/l 30 min after 1 µg Synacthen injection – all included HD patients showed sufficient adrenal response after low-dose Synacthen stimulation with equivalent performance pre- and during HD. Importantly, in 10 of 56 low-dose Synacthen tests (7 tests during HD) the peak cortisol concentration was already reached after 20 min. Thus, in case of low-dose test during HD, an additional measurement of serum cortisol at 20 min can be considered, even though it might not have an influence on the test interpretation.

In our cohort, we did not detect any patients with newly diagnosed adrenal insufficiency, possibly due to the exclusion of patients with ongoing glucocorticoid treatment being the most common cause of adrenal cortical insufficiency [1, 2]. We therefore recommend to perform an initial test during HD, and in case of results suggesting adrenal insufficiency, a confirmatory test off dialysis should be considered. Future research should confirm our results also in other HD patient populations such as in patients in the intensive care setting, during continuous renal replacement therapy, in patients under long-term treatment with low-dose glucocorticoids and in a population with already known adrenal insufficiency. Validating equivalence of Synacthen test pre- and during HD in the above-named patient populations would possibly – in case of results suggesting adrenal insufficiency – make confirmatory test off dialysis redundant.

In our study, we defined sufficient adrenocortical responsiveness as an increase of serum cortisol concentration above 500 nmol/l 60 min after standard-dose (250 µg) and 30 min after low-dose (1 µg) Synacthen, which is in line with the international guidelines of the endocrine society [14–16]. However, pre- as well as during HD it is crucial to consider factors that might influence cortisol concentration and thus interpretation of adrenal function test results: Since glucocorticoids are transported in the blood by corticosteroid-binding globulin (CBG) and albumin, these factors especially include conditions with increased or decreased CBG or albumin such as nephrotic syndrome, liver disease, critical illness, pregnancy or treatment with estrogen-containing oral contraceptives [25–28]. Importantly, in our study there was no hypoalbuminemia and we excluded patients with acute illness, pregnancy or treatment with oral contraception. Unfortunately, we did not measure CBG.

As a secondary aim, we studied the variation of serum cortisol concentrations during an HD session without Synacthen stimulation. Cortisol concentrations at different HD timepoints (30, 60 and 240 min) were significantly different compared to cortisol concentrations at HD start, while a stepwise decrease in cortisol values during the first 60 min after HD start, followed by an increase until the end of the HD session was observed. Patients with HD in the afternoon showed lower cortisol values than patients with HD session in the morning, probably explained by the diurnal variation of cortisol production. However, the behaviour pattern of cortisol levels during HD was similar regardless of the time of HD. Potential factors influencing serum cortisol levels during HD are clearance and metabolism of ACTH and cortisol, hemoconcentration due to volume removal (ultrafiltration), and increased cortisol secretion due to stressful stimuli (such as changes of hemodynamics or plasma glucose). As mentioned above, the role of hemoconcentration seems almost negligible in our study. Glucocorticoids are transported in the blood by CBG and by albumin, whereas CBG – binding about 85% of plasma cortisol – is the main determinant of circulating plasma cortisol levels [29]. Importantly, our study demonstrated no association of serum albumin at dialysis start with cortisol concentrations during HD. Despite the small fraction of unbound (free) cortisol in the circulation, successive removal of free cortisol during HD followed by release of cortisol from protein-bound fraction might partly explain the decrease in cortisol values. After initial decrease, cortisol concentrations increased between 60 min and the end of HD, thus changes of cortisol during HD are not completely explained by dialysance of free cortisol. This is in line with previous data showing that plasma cortisol values tended to be elevated during the second half of a dialysis session [23]. Moreover, a study of radioactive labelled cortisol in seven patients on chronic HD revealed low dialysance values of plasma cortisol – potentially explained by tight protein binding – reaching a plateau at about 60 min of HD [23]. Plasma clearance rates during HD were 30–63% higher compared to off dialysis, while dialysance values accounted only for 20–35% of this increased plasma clearance rate [23]. Thus, besides loss of cortisol into dialysate, other factors must influence plasma clearance of cortisol during HD, such as changes in cortisol metabolism itself, counterregulatory ACTH secretion followed by increased cortisol production. Another potential explanation for the increase in cortisol values until the end of HD might be the increased secretion of arginine vasopressin (AVP) upon changes of blood volume and pressure [30], since AVP induces secretion of ACTH and cortisol [31]. Further investigations to validate these findings with additional measurement of unbound (free) cortisol, CBG and ACTH are still warranted to determine underlying pathophysiology of variation of serum cortisol concentrations during an HD session.

The main strength of our study is that this is the first study investigating the effect of ongoing HD on the adrenal response to exogenously administered ACTH, providing novel insights into the diagnosis of adrenal insufficiency in chronic HD patients. Further strengths are the well-characterized cohort of chronic HD patients, the high statistical power and the direct impact on patient care. The following limitations require consideration: first, we did not detect any newly diagnosed adrenal insufficiency, thus equivalence of Synacthen test pre- and during HD in this patient population could not be examined and the reliability of adrenal function test during HD to detect adrenal insufficiency cannot be proved. Nevertheless, based on our data we think that Synacthen test during ongoing HD should be reliable in the presence of adrenal insufficiency, since potential confounding factors during HD such as clearance of ACTH and cortisol as well as hemoconcentration due to volume removal (ultrafiltration) are expected to be comparable in this patient population. Second, we did not measure levels of ACTH, unbound (free) cortisol or CBG, which might have provided more profound insights into the changes of serum cortisol during HD.

In conclusion, we found equivalent performance of Synacthen test pre- and during HD both for standard- and low-dose Synacthen. Cortisol concentrations 20 and 30 min after low-dose Synacthen administration were shown to be similar. Cortisol concentrations changed significantly during an HD session without Synacthen stimulation, whereas the underlying physiological aspects of these changes are still poorly understood and were not the main subject of this study. The present results suggest that the adrenal function test may be carried out during ongoing HD, leading to a more patient-friendly performance of the test, less organizational effort and thus more accessible diagnostic approach to adrenal insufficiency.

.

### Electronic supplementary material

Below is the link to the electronic supplementary material.

Supplementary Material 1

## Data Availability

All data generated or analysed during this study are included in this published article and its supplementary information files.

## Abbreviations

HD: hemodialysis
