# Supplementary material for: Performance of synacthen test in chronic hemodialysis patients
Source: BMC Nephrol. 2023 Nov 7;24:330. doi: 10.1186/s12882-023-03347-3 (PMC10631216; doi:10.1186/s12882-023-03347-3)

**Supplementary tables and figures**

**Supplementary Table 1:** Estimated effects of patient and HD variables on cortisol concentration on log-scale after Synacthen administration from a multivariable linear mixed model used for equivalence testing. The random intercept has accounted for multiple cortisol measurements for each subject.

**Supplementary Table 2**. Effect estimates of patient and HD variables after low-dose Synacthen administration on cortisol concentration on log-scale at 20, 30 and 60 minutes from a multivariable linear additive mixed model for serum cortisol concentration (nmol/l). Time after Synacthen was fitted with a restricted cubic splines.

Supplementary Table 3: Equivalence test results for cortisol concentration (nmol/l) on log-scale shown in four different Syancthen dose and time settings corrected for hemoconcentration on log-scale. Estimates were fitted in a multivariable linear mixed model with random intercepts per subject accounting for multiple measurement at different time points after Synacthen. It was further adjusted for patient and HD variables: cortisol concentration shortly before Synacthen administration, albumin at HD start on study day 1, glucose after Synacthen, daytime of HD, test sequence and study visit.

**Supplementary Table 4.** Effect estimates from univariate and a multivariable linear additive mixed models for serum cortisol concentration (nmol/l) corrected for hemoconcentration on log-scale during HD without Synacthen stimulation. Multiple measurements during HD per subject were accounted for including a random intercept for subjects. Corrected serum cortisol concentration is modelled on the log-scale accounting for patient and HD variables. Time during HD is assumed to be non-linear and fitted as restricted cubic splines.

**Supplementary Figure 1.** Study plan for each participating patient

**Supplementary Figure 2.** Changes in serum cortisol concentration corrected for hemoconcentration during HD without Synacthen stimulation

**Supplementary Table 1:** Estimated effects of patient and HD variables on cortisol concentration on log-scale after Synacthen administration from a multivariable linear mixed model used for equivalence testing. The random intercept has accounted for multiple cortisol measurements for each subject.

|  | **Effect estimate (95% CI)**  **[p-value]** |
| --- | --- |
| **Cortisol concentration shortly before Synacthen (log-scale)** | 0.298 (0.211, 0.384) **[<0.001]** |
| **Albumin (g/l)*** | -0.001 (-0.020, 0.018) [0.91] |
| **Glucose (mmol/l) after Synacthen** | -0.004 (-0.012, 0.005) [0.41] |
| **Daytime of HD**   - morning - afternoon | (reference)  0.127 (-0.01, 0.265) [0.08] |
| **Synacthen dose**   - low-dose - standard-dose | (reference)  0.028 (-0.020, 0.077) [0.26] |
| **Time of Synacthen administration**   - during HD - pre-HD | (reference)  0.024 (-0.025, 0.073) [0.33] |
| **Time after Synacthen administration**   - 30 min - 60 min | (reference)  -0.245 (-0.293, -0.197) **[<0.001]** |
| **Study day **** | 0.002 (-0.009, 0.013) [0.77] |
| **Sequence *****   - ADBC - BACD - CBDA - DCAB | (reference)  -0.024 (-0.212, 0.164) [0.80]  -0.007 (-0.194, 0.181) [0.94]  -0.046 (-0.246, 0.153) [0.65] |
| **Interaction terms ******   - Time of Synacthen administration x Synacthen dose - Time of Synacthen administration x HD daytime - Synacthen dose x HD daytime - Time of Synacthen administration x Synacthen dose x HD daytime | [0.78]  [0.067]  **[<0.001]**  [0.257] |
| **Between-subject variability (σ_b_)**  **Within-subject variability (σ_ε_)** | 0.172 (0.12, 0.2)  0.092 (0.08, 0.1) |

Abbreviations: CI – confidence interval; HD – hemodialysis; SE – standard error

A – pre-HD, standard-dose; B – during HD, standard-dose; C – pre-HD, low-dose; D – during HD, low-dose

* Measured at HD start on study day 1

** Model variable ‘Study day’ demonstrated that a carry-over effect could not be shown (p>0.05).

*** According to the randomisation protocol, each study participant was assigned to one of the four sequences of possible combinations of Synacthen dose and time of administration. An ‘order’ effect has not been proven (p>0.05).

**** Synacthen dose, time of administration and daytime of HD as main effect and their triple interaction effect have been included into the model to allow to estimate means of cortisol concentration at 30 and 60 minutes after two different Synacthen doses (low, standard) in two different settings (pre-and during HD).

**Supplementary Table 2**. Effect estimates of patient and HD variables after low-dose Synacthen administration on cortisol concentration on log-scale at 20, 30 and 60 minutes from a multivariable linear additive mixed model for serum cortisol concentration (nmol/l). Time after Synacthen was fitted with a restricted cubic splines.

|  | **Effect estimate (SE)**  **[p-value]** |
| --- | --- |
| **Cortisol concentration shortly before Synacthen (log-scale)** | 0.28 (0.155, 0.405) [**p<0.001**] |
| **Albumin (g/l)** | -0.001 (-0.0215, 0.0192) [0.91] |
| **Glucose (mmol/l)** | -0.0126 (-0.027, 0.0015) [0.081] |
| **Daytime of HD**   - morning - afternoon | (reference)  0.124 (0.025, 0.274) [0.105] |
| **Time of Synacthen administration**   - during HD - pre-HD | (reference)  0.045 (0.013, 0.078) [**0.007**] |
| **Time after Synacthen administration**   - **20 min** - **30 min** - **60 min** | -0.02 (-0.1, 0.06) [0.61]  (reference)  0.07 (-0.01, 0.14) [0.09] |
| **Interaction term: Time after Synacthen administration by Synacthen administration time**   - pre-HD:   - 20 min   - 30 min   - 60 min - during HD:   - 20 min   - 30 min   - 60 min | -0.05 (-0.1, 0.002)  (reference)  -0.18 (-0.23, -0.12)  -0.03 (-0.082, 0.021)  (reference)  -0.24 (-0.3, -0.19) |
| **Study day *** | -0.003 (-0.022, 0.017) [0.79] |
| **Sequence ****   - ADBC - BACD - CBDA - DCAB | (reference)  -0.045 (-0.248, 0.158) [0.66]  -0.034 (-0.238, 0.169) [0.74]  -0.071 (-0.289, 0.148) [0.53] |
| **Between-subject variability (σ_b_)**  **Within-subject variability (σ_ε_)** | 0.184 (0.135, 0.252)  0.102 (0.09, 0.115) |

Abbreviations: CI – confidence interval; HD – hemodialysis.

* Model variable ‘Study day’ demonstrated that a carry-over effect could not be shown (p>0.05).

** According to the randomisation protocol, each study participant was assigned to one of the four sequences of possible combinations of Synacthen dose and time of administration. An ‘order’ effect has not been proven (p>0.05).

Supplementary Table 3: Equivalence test results for cortisol concentration (nmol/l) on log-scale shown in four different Syancthen dose and time settings corrected for hemoconcentration on log-scale. Estimates were fitted in a multivariable linear mixed model with random intercepts per subject accounting for multiple measurement at different time points after Synacthen. It was further adjusted for patient and HD variables: cortisol concentration shortly before Synacthen administration, albumin at HD start on study day 1, glucose after Synacthen, daytime of HD, test sequence and study visit.

| **Synacthen dose** | **Time after Synacthen administration (min)** | **Time of Synacthen administration** | **Mean (SE) of cortisol concentration on log-scale** | **Difference in means of cortisol concentration on log-scale**  **pre- vs. during HD**  **[90% CI]** |
| --- | --- | --- | --- | --- |
| Low | 30 | pre-HD | 6.700 (0.040) | 0.0554 [0.015, 0.096] |
|  |  | during HD | 6.644 (0.039) |  |
|  | 60 | pre-HD | 6.531 (0.040) | 0.1305 [0.090, 0.171] |
|  |  | during HD | 6.401 (0.040) |  |
| Standard | 30 | pre-HD | 6.727 (0.040) | 0.0633 [0.023, 0.104] |
|  |  | during HD | 6.664 (0.040) |  |
|  | 60 | pre-HD | 6.878 (0.040) | 0.086 [0.046, 0.126] |
|  |  | during HD | 6.792 (0.040) |  |

**Supplementary Table 4.** Effect estimates from univariate and a multivariable linear additive mixed models for serum cortisol concentration (nmol/l) corrected for hemoconcentration on log-scale during HD without Synacthen stimulation. Multiple measurements during HD per subject were accounted for including a random intercept for subjects. Corrected serum cortisol concentration is modelled on the log-scale accounting for patient and HD variables. Time during HD is assumed to be non-linear and fitted as restricted cubic splines.

| **Effect estimate (95% CI)**  **[p-value]** | **Univariate** | **Multivariable** |
| --- | --- | --- |
| *Patient variables at start of HD* | | |
| **Age (years)** | 0.004 (-0.007, 0.014) [0.48] | - |
| **Gender:**   - Male - Female | (reference)  -0.06 (-0.29, 0.17) [0.60] | - |
| **Dry weight (kg)** | 0.002 (-0.003, 0.006) [0.52] | - |
| **Albumin (g/l)** | 0.010 (-0.018, 0.037) [0.49] | 0.010 (-0.014, 0.034) [0.41] |
| *Patient variables varying over HD time* | | |
| **Loss of weight between start and end of HD (kg)** | -0.063 (-0.149, 0.022) [0.16] | - |
| **Glucose (mmol/l)** | 0.013 (-0.019, 0.044) [0.44] | -0.007 (-0.03, 0.02) [0.61] |
| **Systolic blood pressure*** | -0.003 (-0.006, 0.0002) **[0.07]** | -0.004 (-0.007, -0.001) **[0.005]** |
| **Diastolic blood pressure*** | 0.001 (-0.004, 0.007) [0.63] | - |
| **Daytime of HD:**   - Morning - Afternoon | (reference)  -0.29 (-0.47, -0.11) **[0.004]** | (reference)  -0.28 (-0.46, -0.10) **[0.004]** |
| **Ultrafiltration amount** | -0.023 (-0.05, 0.003) [0.09] | - |
| **HD time (min)**   - 0 - 30 vs. 0 - 60 vs. 0 - 240 vs. 0 | (reference)  -0.21 (-0.28, -0.14)  -0.39 (-0.51, -0.26)  -0.40 (-0.53, -0.27)  **[<0.001]** | **-** |
| **HD time (min) at morning:**   - 0 - 30 vs. 0 - 60 vs. 0 - 240 vs. 0   **HD time (min) at afternoon:**   - 0 - 30 vs. 0 - 60 vs. 0 - 240 vs. 0 |  | (reference)  -0.18 (-0.29, -0.07)  -0.34 (-0.54, -0.14)  -0.52 (-0.72, -0.32)  **[<0.001]**  (reference)  -0.21 (-0.31, -0.11)  -0.39 (-0.57, -0.21)  -0.32 (-0.50, -0.15)  **[<0.001]** |
| *Random effects* | | |
| **Between-subject variability (σ_b_)**  **Within-subject variability (σ_ε_)** |  | 0.19 (0.12, 0.31)  0.25 (0.21, 0.30) |

**Supplementary Figure 1.** Study plan for each participating patient

**
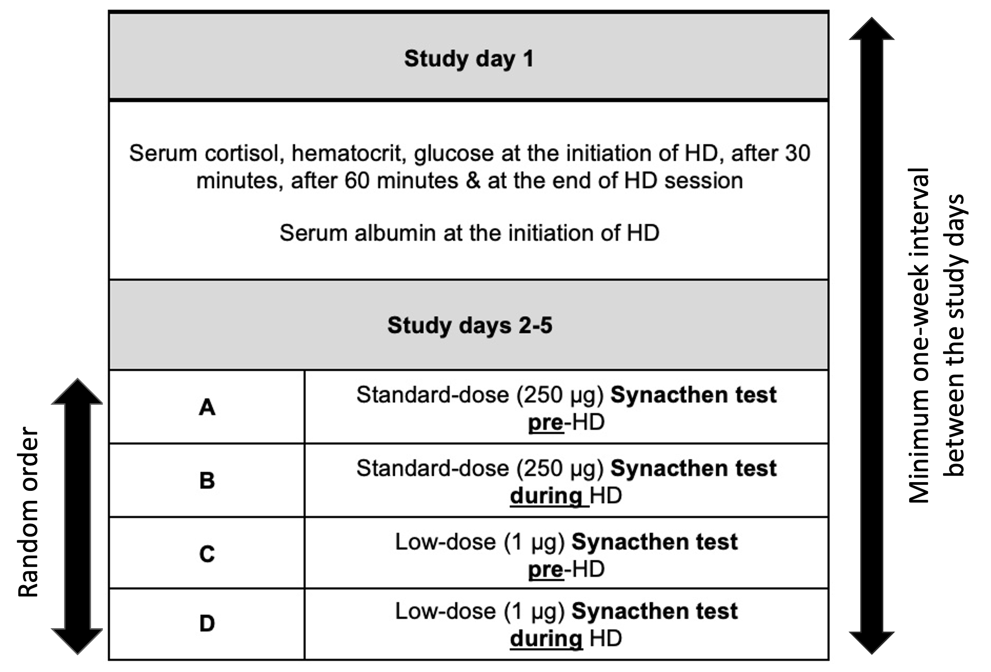
**

**Supplementary Figure 2.** Changes in serum cortisol concentration corrected for hemoconcentration during HD without Synacthen stimulation


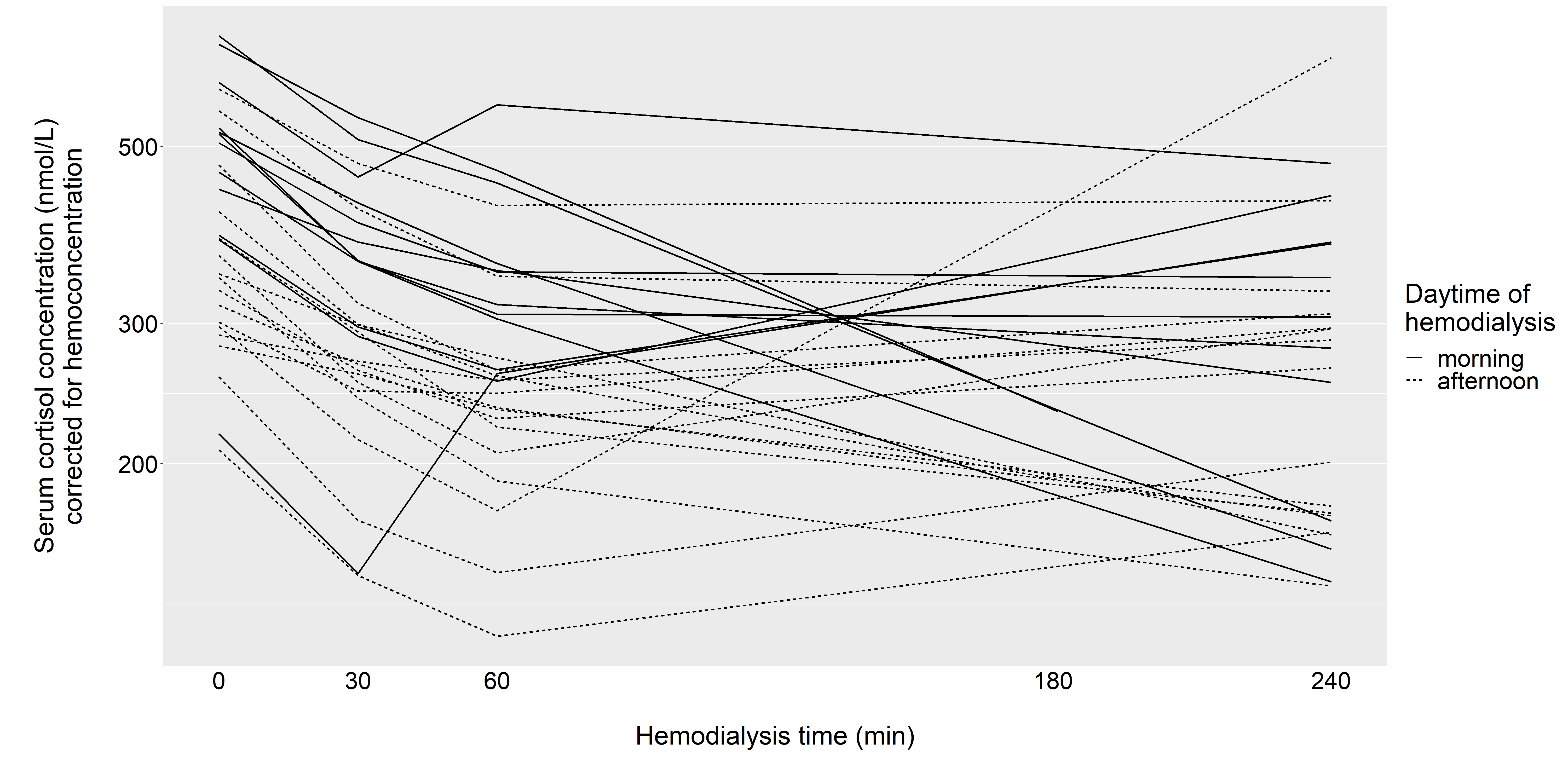

Supplement: Supplementary file 1 — Supplementary Material 1 [file 12882_2023_3347_MOESM1_ESM.docx]
